# Lymph node status as a guide to selection of available prognostic markers in breast cancer: the clinical practice of the future?

**DOI:** 10.1186/1746-1596-1-41

**Published:** 2006-11-08

**Authors:** A Elzagheid, T Kuopio, S Pyrhönen, Y Collan

**Affiliations:** 1 Department of Oncology and Radiotherapy, Turku University Hospital, Savitehtaankatu 1 PB 52, FIN-20521, Turku, Finland; 2 Department of Pathology, University of Turku, and Turku University Hospital, Kiinamyllynkatu 10, FIN-20520, Turku, Finland; 3 Department of Pathology, Jyväskylä Central Hospital, FIN-40620, Jyväskylä, Finland

## Abstract

Prognosticators evaluating survival in breast cancer vary in significance in respect to lymph node status. Studies have shown e.g. that HER2/neu immunohistochemistry or HER2/neu gene amplification analysis do perform well as prognosticators in lymph node positive (LN +) patients but are less valuable in lymph node negative (LN -) patients. We collected data from different studies and tried to evaluate the relative significance of different prognosticators in LN+/LN- patient groups. In LN+ patients HER2/neu and E-cadherin immunohistochemistry were the statistically most significant prognosticators followed by proliferation associated features (mitotic counts by SMI (standardised mitotic index) or MAI (mitotic activity index), or S-phase fraction). Bcl-2 immunohistochemistry was also significant but p53 and cystatin A had no significance as prognosticators. In LN- patients proliferation associated prognosticators (SMI, MAI, Ki-67 index, PCNA immunohistochemistry, S-phase fraction) are especially valuable and also Cathepsin D, cystatin A, and p53 are significant, but HER2/neu or bcl-2, or E-cadherin less significant or without significance. We find that in studies evaluating single prognosticators one should distinguish between prognosticators suitable for LN+ and LN- patients. This will allow the choice of best prognosticators in evaluating the prospects of the patient. The distinction between LN+ and LN- patients in this respect may also be of special value in therapeutic decisions.

## Background

Breast cancer is the most common cancer among women and the second leading cause of cancer deaths in women today. For example, 212,920 new cases of invasive breast cancer are expected to be diagnosed among women in the United States in 2006 (1.4 new cases among 1000 women) [1]. In Finland, according to the Finnish Cancer Registry, about 3800 females are diagnosed with breast cancer every year (1.5 new cases among 1000 women)[2]. Improved methods of detection and treatment of breast cancer have had a significant influence on disease outcome [3]. The main challenge today is to find factors which could predict the patients who have a tumor with aggressive nature. There are a lot of data on clinical prognostic factors such as axillary lymph node (LN) status, tumor size, histological grade, and clinical stage. Breast cancer treatment could be based on these factors [4]. However, additional factors have also been used such as estrogen receptor (ER) positivity, progesterone receptor (PR) positivity, and HER2/neu status. The latter are considered especially useful in identifying patients who benefit from systemic adjuvant therapy. The International Consensus Panel of St. Gallen determined the standard prognostic factors of breast cancer as follows: LN status, ER and PR, tumor size, histological grade, and age [5]. These prognostic factors have been used to identify the high-risk patients who may benefit from the adjuvant systemic therapy. However, still there is an urgent need for finding biological factors which could help in planning the future therapy of breast cancer.

Here we review several biological prognostic factors and associate their prognostic role with the lymph node status. Such grouping could help clinicians to create tailored and more individualized prognostic models which could help in choosing the right treatment for individual patients. Earlier research has shown that prognosticators vary between LN+ and LN- patients. Table 1 shows the characteristics of a few studies we will refer to in the following.

**Table 1:** Survival associated tissue-section-based prognostic factors and lymph node status in breast cancer.

**Prognosticator**	**N**	**Age range or median**	**Country**	**Methods**	**Stage/LN status of all patients**	Stength of association (survival %)*	**P value, type of analysis**	**Mean or median follow-up (months)**	**Reference**
**LN+ patients**									
AgNOR	164	N.A.	Italy	MA	N-, N+	DFS (42,61)	0.0093 M	108	Derenzini^90^
Bcl-2	107	56	Belgium	IHC	N-, N+	DFS (21,62)	<0.001 U	91	Hellemans^55^
CD44	74	62	Finland	IHC	N-, N+	DSS (45,56)	0.02 U	84	Joensuu^158^
E-cadherin	57	59	Finland	IHC	N-, N+	OS (36,88)	0.0001 U	66.5	Elzagheid^150^
EGFR	404	53.4	Japan	IHC	N-, N+	DFS (50,75)	<0.0001 U	46	Tsutsui^110^
ER	705	54	UK	IHC	I-IV	DFS, ReR 0.651	0.004 M	62	Rehim^122^
HER2/neu	106	59	Finland	IHC	N-, N+	OS (13,80)	0.001 U	67.2	Jalava^16^
IGF-1	98	57.1	Finland	IHC	N-, N+	OS (16,35)	0.0286 U	169.2	Toropainen^115^
Mitoses	131	59	Finland	SMI	N-, N+	RR 3.5	0.0005 U	69	Kronqvist^71^
Mitoses	368	25–81	MC	MAI	N+	OS (57,83)	0.00003 U	60	Simpson^68^
P21	328	N.A.	USA	IHC	N-, N+	DFS, X^2 ^74.61	0.054 M	195.6	Thor ^37^
PgR	106	58	Finland	IHC	N-, N+	N.A.	0.0186	70	Jalava ^123^
SPF	257	25–81	MC	FCM	N+	OS (70,82)	0.026 U	60	Simpson^68^
									
**LN- patients**									
Ploidy	50	56	UK	FCM	N-	DFS (32,71)	<0.0001 U	>120	Yuan^132^
Cathepsin D	262	60	Finland	IHC	N-	DFS (47,68)	<0.0001 U	98.4	Isola^100^
CD44	237	56	Holland	IHC	N-, N+	DFS (50,75)	0.005 U	84	Foekens^156^
EGFR	618	53.4	Japan	IHC	N-, N+	DFS, ReR 2.05	0.0241 M	46	Tsutsui^110^
HER2/neu	324	N.A.	USA	FISH	N-	DFS (57,75)	0.0077 U	50	Press^17^
HER2/neu	224	59	Finland	IHC	N-, N+	N.A.	N.S.	67.2	Jalava^16^
Ki-67	89	60.5	Finland	IHC	N-, N+	OS (50,70)	0.0297 U	103.2	Pietilainen^78^
Mitoses	516	<55	MC	MAI	N-	DFS (62,85)	<0.00001U	118	Baak^69^
Mitoses	232	59	Finland	SMI	N-, N+	RR 4.2	0.0007 U	69	Kronqvist^71^
P27	75	<65	Canada	IHC	N-, N+	DSS, ReR 0.24	0.03 M	180	Foulkes^47^
P53	700	<50	USA	IHC	N-	DFS (53,85)	0.0001U	54	Allred^33^
PCNA	205	60.5	Finland	IHC	N-	DFS (56,80)	0.0003 U	141.6	Aaltomaa^84^
SPF	180	N.A.	China	N.A.	N-	N.A.	<0.001	60	Zhang ^96^

Prognosticators for LN+ patients are presented first followed by those for LN- patients. The database applied was PubMed, the presented papers were published during years 1991–2005. The most significant p-values with defined survival characteristics for each marker are shown. P-values are not comparable with each other in absolute terms because also the size of the sample influences the p value. The value of prognosticators can only be compared reliably by comparing different prognosticators in the same patient material.

* The figures within the parantheses refer to the survival of 2 cutpoint-associated patient groups after median follow up. IHC, immunohistochemistry; N-, node negative; N+, node positive; MA, morphometric analysis; FCM, flow-cytometry; NA, not available; MC, multicenter study; SMI, standardized mitotic index; MAI, mitotic activity index; OS, overall survival; DFS, disease free survival; DSS, disease specific survival; RR, risk ratio; ReR, relative risk; X^2^, Chi square;, FISH, fluorescence in situ hybridization; N.S, not significant; M, multivariate; U, univariate.

Differences in the size and other characteristics of the studied populations make comparison between different reports difficult. Most of the studied populations are Caucasian (white), but in two American studies, there is also a fraction of black patients [37,68]. Because populations may differ in prognostic characteristics, we have decided to stress this point [1,65]. Most studies do not include adjustment for age and there are studies which do not necessarily clearly describe how cutpoints for each prognostic marker were defined. [In writing this review, however, we have expected that the authors have been able to select the most significant cutpoint for each prognosticator studied (see e.g. [123])].

### Oncogenes and tumor suppressor genes

#### C-erbB-2 (HER2) or HER2/neu

HER2/neu proto-oncogene encodes a 185-KDa transmembrane glycosylated epidermal growth factor receptor that contains an extracellular domain and has intracellular tyrosine kinase activity [6]. Amplification of the HER2/neu gene and overexpression of its protein are known to be characteristic of many breast cancers [7-9]. Several studies have correlated the overexpression of the HER2/neu oncogene with poor prognosis in breast cancer patients [10,11], and demonstrated that evaluating HER2/neu protein by immunohistochemistry (IHC), fluorescence in situ hybridization (FISH), and chromogenic in situ hybridization (CISH) are important in selecting optimal therapy and predicting prognosis in breast cancer patients [12-14]. Slamon et al. and Jalava et al. [15,16], showed that intensive HER2/neu immunostaining was a highly significant negative prognosticator in LN+ patients (especially postmenopausal), but not a significant prognosticator among LN- patients or patients younger than 52 years. The paper of Jalava et al. [16] suggested that the size of the patient group which absolutely seemed to need the targeted therapy against the amplified HER2/neu receptor was at least 2% of all breast cancer patients. Press et al. [17], however, suggested that HER2/neu is a significant prognosticator in LN- patients. However, there is still no perfect consensus on which method is most predictive of positive patient response to trastuzumab, also known as Herceptin, a monoclonal antibody that selectively targets HER2/neu. Tawfik et al. [18] discussed the results from a study comparing HER2/neu expression and gene amplification in the same patients by IHC using automated cellular imaging system (ACIS) and by FISH. They concluded that HER2/neu assessment by IHC-ACIS correlates highly with results obtained by FISH. The concordance rate between two methods was 94%. Isola et al. [19] showed that CISH could provide an accurate and practical alternative to FISH for clinical diagnosis of HER2/neu oncogene amplification in archival formalin-fixed breast cancer samples. Seidman and associates [20] reported that the overall response rate (for combination paclitaxel and trastuzumab) in patients with HER2/neu overexpression ranged from 67% to 81%, compared with 41% to 46% in patients with tumors having normal Her-2 expression. In agreement with them Joensuu et al., Piccart-Gebhart et al., and Romond et al. [21-23] demonstrated positive response among patients showing HER2/neu amplification or intensive membrane staining by immunohistochemistry. The effect was clear in disease-free- survival and overall survival in freedom from distant metastases.

#### P53

Early observations after the identification of the p53 protein suggested that p53 functioned as an oncogene. In fact it does, but only in the mutated form. In the late 1980s, however, several discoveries proved that the normal function of p53 was anti-oncogenic. One of the several functions of the normal p53 gene is suppression of cell proliferation. When DNA is damaged p53 inhibits the progression of cell cycle from G1 to the S-phase or during S-phase [24-26]. The mutation of the p53 gene has been detected in almost 50% of human cancers including breast cancer [27-29]. Several studies demonstrated that mutations of p53 or increased nuclear expression of p53 protein is a prognostic factors in breast cancer, and associated with worse prognosis [30-32]. Allred and associates [33] showed that p53 predicted disease free survival in patients with LN- breast cancer. Expression of mutant p53 protein was associated with early disease recurrence and early death in LN- breast cancer. The results of Kuopio et al. [34] clearly support the latter finding. In their series immunohistochemical positivity of p53 was a significant prognosticator among all and LN- patients, but did not show significance among LN+ patients.

#### P21

p21 (WAF) is an inhibitor of cyclin-dependent kinase. It plays a central role both in the regulation of the cell cycle and in the DNA replication [35]. p21 (WAF) is regarded as a putative tumor suppressor and its role in breast cancer is still unclear. p21 (WAF) might have a functional role in the inhibition of PCNA mediated DNA replication [35]. So far there is not much evidence that p21 (WAF) could be used as a prognostic factor in breast cancer [36]. Thor and co-workers demonstrated marginal independent prognostic significance of the p21 (WAF) in the LN+ set of patients, whereas p21 status was not significantly associated with survival among LN- patients [37]. Ras p21 is a guanine nucleotide-binding protein that is involved in the signal transduction pathway that control cell proliferation [38,39]. The ras gene is rarely mutated in breast tumors, but is overexpressed in 50–60% of breast tumors [40]. Rundle et al. suggested that the presence of ras p21 in blood is associated with a five fold increased risk of breast cancer [41]. Czerniak et al. found that p21 expression was significantly higher in cancer cells than in epithelial cells of control specimens. As a group, tumors with axillary lymph node metastases expressed higher levels of ras p21 than nonmetastasizing tumors [42].

#### P27

P27 is a cyclin dependent kinase inhibitor, which may act as a potential suppressor gene. Reduction of p27 expression is related to uncontrolled cell proliferation and tumorigenesis. P27 acts in G1 to inhibit cyclin-cdks [43,44]. Several studies have suggested that low expression of p27 is an independent factor weakly associated with poor prognosis [45,46]. Foulkes and his co-workers (2004) [47] demonstrated that the level of p27 was one of independent predictors in LN- patients but not in LN+ patients.

#### C-myc

Amplification of the c-myc gene is found in 20% of primary breast cancers, and this is more frequent in larger tumors, and in lymph node positive patients [48]. Overexpression and other alterations of c-myc gene may be related to breast cancer progression. It can be expected that c-myc has a potential as a marker of poor prognosis [49,50]. We did not find c-myc associated prognostic information which was related to the lymph node status.

#### Bcl-2

Bcl-2 gene encodes for a mitochondrial protein thought to prevent apoptosis in normal cells. Dysregulation of this gene can contribute to tumor progression and increased drug resistance [51,52]. Bcl-2 expression was associated with favourable prognosis in breast cancer and was both estrogen receptor(ER) and progesterone receptor (PR) associated [53,54]. Several studies have demonstrated the independent favourable prognostic impact of Bcl-2 on breast cancer particularly among LN+ patients [55,56]. It is obvious that the prognostic value of Bcl-2 staining among all patients is solely based on prognostic applicability among LN+ patients because the association among LN- patients is clearly non significant [57].

### Cell proliferation

#### Mitotic count

Evaluation of proliferation activity of the neoplastic cells gives important prognostic information, especially in breast cancer [58,59]. There are many available ways to measure cell proliferation, including the determination of the mitotic rate by counting the mitotic figures [60,61]. Mitotic counts are performed by counting the number of mitoses from ten high power fields (Baak et al. ; mitotic activity index; MAI), or by expressing the count by square millimeter, which produces the standardised mitotic index (SMI) [62], or volume fraction corrected mitotic index (M/V_v _index) [63,64]. Many studies demonstrated that the MAI is an independent prognostic factor for recurrence free survival [65,66]. SMI is a bit more efficient than MAI as a prognosticator [67]. Simpson et al. [68] showed that determination of mitotic activity is able to identify a group with improved disease free and overall survival among patients with LN+ breast cancer. The mitotic activity is also an independent prognostic marker in LN- breast cancer patients younger than 55 years [69]. Baak and his co-worker also found that the LN- patients with MAI > 10 are at risk for distant metastases, similar to LN+ [70]. SMI was a highly significant prognosticator among all patients but especially among LN- patients [71]. Among LN+ patients this marker was less significant. It is surprising in light of numerous ways to measure proliferative activity how consistently the mitotic count emerges as the most powerful prognosticator. All ways of estimation are prognosticators but the mitotic count is the strongest one in the majority of comparing studies.

#### Ki-67

Ki-67 antigen is one of several cell-cycle regulating proteins which can be determined by immunohistochemistry [72,73]. It is expressed in proliferating G1-, S- and G2/M-phase nuclei [74,75]. Because most of the Ki-67 positive cells are in the cell cycle, Ki-67 labelling index (fraction of Ki-67 positive nuclei of all nuclei) reflects the fraction of proliferating cells. Many studies demonstrated the association between a high Ki-67 labelling index, histological grade and large tumor size in breast carcinoma [76,77]. Ki-67 labelling index (determined e.g. by the MIB1 antibody; an IgG monoclonal antibody used for detection Ki-67) has prognostic value in breast cancer, particularly in LN- patients [78,79]. However, the prognostic significance is less than that of mitotic count (as determined with either MAI or SMI). However, there is some evidence that 3-dimensional analysis of Ki-67-positive nuclei may improve the prognostic power of Ki-67 immunohistochemistry [80].

#### PCNA

Proliferating cell nuclear antigen (PCNA) is a DNA-polymerase-related protein which is expressed in all proliferating cells. The expression is increased during G1 phase, S-phase and declines during G2/M [81]. Many reports observed that PCNA shows prognostic value in breast cancer [82,83]. Aaltomaa and his coworkers stated that the prognostic significance of PCNA can be demonstrated especially among LN- patients [84]. They did not include LN+ patients in their study.

#### AgNOR

Argyrophilic nucleolar organizer regions (AgNOR) correlate with the proliferative activity of neoplasms. Increased AgNOR counts may reflect increased proliferative activity of cells [85,86]. AgNOR counts have been studied in breast carcinoma, but the results have been conflicting. Some studies have demonstrated that quantitative analysis of AgNORs yields a prognostic factor in breast cancer [87,88]. The combination of MIB-1- immunopositivity and AgNOR measurements in MIB-1 positive nuclei improves prognostication [89]. Derenzini et al. reported that AgNOR has a prognostic value specially among LN+ patients [90]. Others were not able to find prognostic significance in AgNOR counts for breast cancer [91,92].

#### S-phase fraction (SPF)

The histogram describing the nuclear DNA content determined by flow cytometry can be used as an estimate of the proliferation rate. The measurement estimates the fraction of cells in the S phase (S phase fraction), which reflects proliferative activity. The S-phase fraction (SPF) is a rough estimate of neoplastic growth rate. Several reports observed the prognostic value of SPF in breast cancer [93,94]. Low SPF is associated with an excellent prognosis in LN- breast patients [95-97]. Based on the grading efficiency analysis [98] SPF estimation from isolated nuclei may reach efficiencies comparable to that of mitotic counts.

### Cathepsin D (CD)

Cathepsin D is a 52-kd protein, precursor to a lysosomal acidic protease. This proteolytic enzyme can degrade basement membranes. It has mitogenic activity on MCF-7 cells that are estrogen depleted and contributes to proliferation, invasion, and progression in breast [99]. Several studies have demonstrated that Cathepsin D is an important prognostic factor in breast cancer, especially in LN- patients [100,101]. In the report by Rochefort [102] overexpression of cathepsin D was associated with increased risk of metastasis, but there are also studies that failed to find prognostic significance of cathepsin D in breast cancer [103,104].

### Growth factors

#### Epidermal growth factor receptor (EGFR)

EGFR is a member of the tyrosine kinase growth factor receptor family. EGFR and its ligand, transforming growth factor-alpha (TGF-alpha) play an important role in several human cancers [105,106], through the autocrine growth-regulation system in breast cancer, EGFR has been reported to be associated with a poor clinical outcome [107,108]. Rampaul and his co-workers found that the EGFR was significant only in lymph node positive breast cancer patients [109], with high expression reflecting worse outcome. Tsutsui et al. reported that a combination of EGFR and ER was an independent significant factor for both disease free survival (DFR) and overall survival (OS) both in patients with LN- and LN+ breast cancer. Patients with EGFR (+) and ER (-) had worse DFS and OS [110].

#### Insulin-like growth factor (IGF)

IGF1 and IGF2 are circulating peptide hormones and locally acting growth factors with both paracrine and autocrine functions. Both IGF1 and IGF2 are involved in the regulation of cell proliferation and apoptosis [111]. Many observers suggested that IGFs are involved in the progression of breast cancer [112,113]. The presence of IGF-1 immunoreactivity in breast cancer epithelial cells indicates a lower degree of malignancy than the lack of IGF1 [114]. Toropainen et al. reported that, in a univariate analysis, IGF-1 was significantly related to a high survival probability particularly in LN+ breast cancer patients [115].

#### Estrogen (ER) and progesterone (PR) receptors

Since breast cancer is one of the hormone dependent tumors much attention has been paid to the relationship between ER and PR and breast cancer. The study by Blanco et al. (1984) [116] showed that ER+ PR+ patients had better prognosis than ER- PR- patients. In late 1970's and early 1980's the measurement of ER as well as PR became standard practice in the prediction of the outcome of breast cancer patients [117-119]. The immunohistochemical analysis of ER and PR has replaced the traditional and also clinically validated dextran charcoal radioactive ligand binding assay [120]. The prognostic value of ER and PR seems to be greater among axillary LN+ than among LN- patients [121,122]. The study of Jalava et al. [123] showed that immunohistochemical ER score is associated with prognosis. However, cutpoints for defining the groups with good or worse prognosis may differ between LN- and LN+ patients.

### DNA content

Cytometric quantitation of nuclear DNA content can assist in the diagnosis and grading of malignant tumors [124]. A great number of studies using flow cytometry and static image cytometry suggested that nuclear DNA content has a significant value in prognosis of breast cancer [125-127]. Tumors with DNA peaks within diploid limits have a more favorable prognosis than those with aneuploid peaks [128,129]. Nuclear DNA content strongly correlated to histopathologic grade of ductal carcinoma. Histologic grade 3 tumors were more likely to be aneuploid than others [130,131]. Many observers stated that aneuploid tumors were associated with a poorer prognosis than diploid tumors in LN- breast cancer patients [132,129]. However, an adverse correlation was stated by Chassevent et al. They confirmed that ploidy status had no prognostic impact in the overall population or in subgroups defined by lymph node status [133]. Moureau- Zabotto et al. suggested that combination of DNA ploidy and SPF predict patients out-come particular in LN- breast cancer patients [134]. DNA ploidy results can be combined with other features in efficient evaluation of prognosis [135].

### Nuclear morphometry

Baak and his coworkers (1985) were among the pioneers in introducing morphometry for prognostication of breast cancer [62]. They found that morphologic features are associated with high risk in pre-invasive breast cancer. The independent prognostic value of nuclear variables was established in several studies on infiltrating breast cancer [136]. Nuclear area and diameter were shown to be useful prognostic factors. However, nuclear diameter has failed to separate the primary tumor from its metastasis [137]. Patients with high nuclear area values and high SD of nuclear area values tend to have poor prognosis [138]. High mean nuclear area is related to poor histological grade [139]. Evaluation of nuclear area can be used for morphometric grading of breast cancer [140]. The latter study also showed that the nuclear area was a significant prognosticator among LN+ and all patients, but completely lacked significance among LN- patients.

### Adhesion molecules

#### E-cadherin

Cell adhesion molecules play an important role in the maintenance of tissue architecture [141]. E-cadherin is a member of a family of transmembrane glycoproteins that mediate homotypic calcium dependent cell-to-cell adhesion in epithelial tissues [142]. Loss of normal function of E-cadherin is known to promote cancer invasion in several human cancers [143]. Several studies have demonstrated that reduced E-cadherin expression is an indicator of increased invasiveness and dedifferentiation in breast cancer [144,145]. A recent study found that the expression of E-cadherin correlates with histological type and grade [146]. The expression of E-cadherin in infiltrative lobular carcinoma was completely negative or weakly positive whereas infiltrating ductal carcinoma showed greater immunoreactivity in grade 1 breast carcinoma than in grade 2 and grade 3 carcinomas [147]. There was an inverse relationship between E-cadherin expression and axillary lymph node involvement. Reduction in E-cadherin expression was associated with the involvement of the axillary lymph nodes [148]. Gamallo and his associates [149], and Lipponen and his co-worders [142] did not agree with this finding. Siitonen et al. [144] showed that reduced E-cadherin expression was independently associated with shorter disease free survival. Other authors stated that the prognostic value of E-cadherin was stronger among patients with LN+ breast cancer [150,151] than among all patients. However, there was no significant association with prognosis among LN- patients [150]. The latter findings can explain the variable findings by different research groups on the prognostic significance of E-cadherin among all breast cancer patients.

#### Catenins

Intracellular proteins (α-β-γ-catenins), are associated with E-cadherin's cytoplasmic tail and link actin to the microfilament network of the cellular cytoskeleton. This binding is essential for the adhesive function of E-cadherin [152]. Alterations in expression of catenins may lead to the disassembly of cadherin junctions and to the generation of more invasive cells [153]. Many studies have suggested that loss of E-cadherin and catenin expression may be associated with poor prognosis of breast cancer [154,155]. We did not find any reports of catenins associated with prognostic data which is related to the lymph node status.

#### CD 44

CD44 is a family of cell surface transmembrane glycoproteins which is expressed in a wide variety of tissues and cell types [156,157]. In human breast cancer, the prognostic value of the various isoforms has not been recognized as independent predictors of breast cancer outcome [158,159]. Foekens and co-workers [156] stated that the expression of exon v6 of CD44 may be a marker for identifying patients with relatively favourable prognosis among LN- patients.

### Extracellular matrix

#### Tenascin

Tenascin is a glycoprotein of the extracellular matrix and appears in the stroma of benign and malignant tumors [160]. Many studies have stated that loss of tenascin expression in breast cancer cells appears to indicate poor prognosis [161,162]. So far we do not have knowledge of differences in the prognostic value between LN + and LN - patients.

#### Matrix metalloproteinases (MMPs)

MMPs are a family of proteinases that play an important role in malignant tumor growth, invasion, metastasis and angiogenesis [163,164]. High expression levels of MMPs in tumor tissue are usually associated with poor survival [165]. In breast cancer, Talvensaari-Mattila and his coworker [166] demonstrated that overexpression of MMP-2 is associated with poor prognosis. Prognostic data on LN + and LN - patients are not available.

### Cystatin A

Cystatin A is a natural cysteine proteinase inhibitor and is found in a wide variety of normal cells. The physiologic role of Cystatin A is not fully known, however. Cystatin A is present in large amounts in follicular dendritic cells in lymphoid tissues [167]. In malignant tissues, cystatin A has been found in many tumors including breast cancer [168]. In breast cancer patients, Kuopio et al. (1998) demonstrated that the expression of cystatin A was associated with poor outcome; the study also showed that cystatin A was a prognostic marker for LN- patients. In LN+ patients, cystatin A was without significance [34].

### Grading

Various histological grading systems of breast carcinoma have been described [169-171]. The majority of tumor grading systems combine nuclear grade, tubular formation and mitotic rate. In Europe, Elston and Ellis (1991) stressed the importance of careful grading [172] and corresponding approach has become increasingly popular in the US [173,68]. It was suggested that the histological grade functions best as a prognosticator in LN- patients for making decision when tumor sizes fall between tumor size categories [174]. The applied histological features can be measured morphometrically [71,140,175], making the grading system robust and reproducible. The multivariate grading methods [62,176-178] are comparable or better than grading methods alone and can be expected to be more reproducible. However, the performance in grouping of the patients in low and high grade categories is not necessarily identical because these grading methods were originally based on different patient materials [179]. It is important to notice that most multivariate methods include lymph node status as a contributing feature and for that reason multivariate methods or indices are applicable to both LN+ and LN- patients. This also applies to the Nottingham prognostic index which combines tumor size, lymph node status, and histological grade [172]. So far, however, there are few studies comparing traditional and multivariate grading in clinical practice or in different patient groups in terms of power to finding patients with bad prognosis. But we know that different individual prognostic factors and grading systems often give parallel results. For example: when DNA ploidy status is diploid, histological grade is generally low, and when DNA ploidy is aneuploid, histological grade is higher [180]. If histological grade is used as the basic classifier of patients into treatment groups, DNA ploidy can be used as a method to confirm the classification especially in situations of uncertainty. In the same way, other biological prognosticators can and probably should be applied in clinical practice.

## Conclusion

Mitotic activity indices (SMI or MAI) are the most powerful general prognostic markers and highly associated with survival in LN+ patients and in LN- patients younger than 55 years old.

E-cadherin was the second most important prognostic marker in patients with LN +, followed by HER2/neu. In LN- patients, cathepsin D was the second most important prognostic marker followed by HER2/neu, SPF, and aneuploidy.

Table 1 lists histological prognostic markers in LN- and LN+ breast cancers. Because the presented survival associated significances are based on different studies, they are not directly comparable. However, reliable comparison in term of prognostic value can be done after univariate analysis in the same group of patients. Multivariate analysis is less suitable for comparison of variables because the goal of the multivariate analysis is the removal of the prognostic overlaps. The strength of association can be evaluated either by risk ratio, relative risk, or comparison of the survival curves defined by 2 cutpoint associated patient groups in respect to disease free survival (DFS), overall survival (OS), or disease specific survival (DSS). The difference in the importance of prognosticators is presented in Table 2. Generally, comparison is made difficult by the lack of standardized study protocols. Table 2 shows, however, that prognosticators vary in terms of stage: the survival of LN- and LN+ patients can best be predicted with prognosticators specific to the LN status.

**Table 2:** Comparison of different prognosticators in single studies on breast cancer.

**LN +**	Prognosticator	P value
Kuopio et al. 1998 [34]		
	1. Bcl-2	0.017
	2. p53	0.121
	3. Cystatin A	0.386
Jalava et al. 2002 [16]		
	1. ErbB2	0.0002
	2. SMI	0.0014
	3. Bcl-2	0.048
Elzagheid et al. 2002 [150]		
	1. E-cadherin	0.0006
	2. SMI	0.0133

**LN -**		

Kuopio et al. 1998 [34]		
	1. Cystatin A	0.010
	2. p53	0.021
	3. Bcl-2	0.874
Jalava et al. 2002 [16]		
	1. SMI	0.0001
	2. ErbB2	N.S.
	3. Bcl-2	N.S.
Elzagheid et al. 2002 [150]		
	1. SMI	0.0299
	2. E-cadherin	0.5581

P values refer to the results of univariate analysis. Comparison of univariate analyses in a study allows one to conclude on the relative strengths of different prognosticators.

We conclude that studies on prognostic evaluation of new potential markers in breast cancer should not be undertaken without studying the prognosis among all patients, and separately among LN + and LN - patients. Correspondingly the use of prognosticators for clinical decision making should be based on the gathered knowledge of differences between LN + and LN - patients.
